# Clinical study on femtosecond laser restoration of accommodation

**DOI:** 10.1007/s00417-023-06188-w

**Published:** 2023-08-07

**Authors:** Omid Kermani, Georg Gerten, Uwe Oberheide, Holger Lubatschowski

**Affiliations:** 1 Artemis Augenklinik am Neumarkt, Schildergasse 107-109, Cologne, 50667 Köln, Germany; 2 Institut für Angewandte Optik und Elektronik-Technische Hochschule TH, Cologne, Germany; 3 ROWIAK GmbH, Hannover, Germany


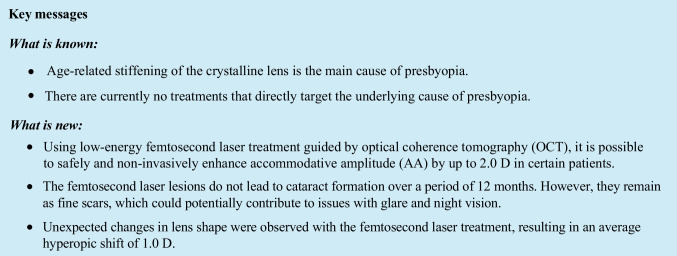


Presbyopia is primarily caused by the progressive stiffening of the crystalline lens due to aging, leading to a gradual decline in accommodative amplitude (AA). While previous studies have focused on various aspects of the condition, such as the lens capsule, zonular fibers, or ciliary muscles, recent research has explored the potential of using femtosecond laser treatment to address presbyopia at its root cause without inducing cataracts [1–5]. A specific femtosecond laser was developed in 2012, specifically designed to make precise cuts for presbyopia treatment. To assess its efficacy in increasing AA, we simulated different laser patterns using a finite element model (FEM). Objective measurements of accommodation were obtained through optical coherence tomography (OCT), refractometry, and wavefront aberrometry. Safety was confirmed in a trial involving 15 cataractous eyes, paving the way for subsequent randomized phase 2a trials.

**Fig. 1:**
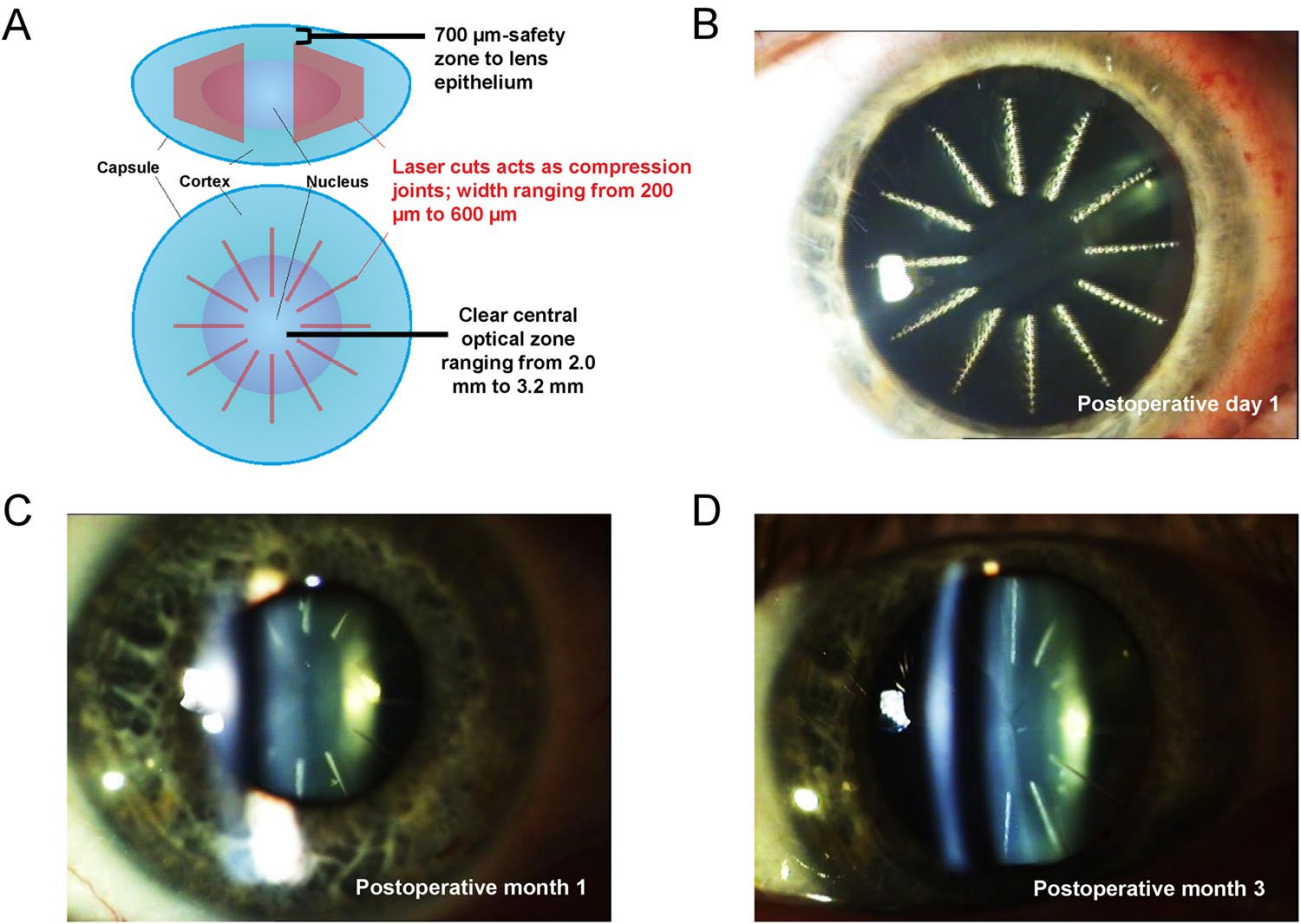
Femtosecond laser treatment of patients with presbyopia. **A** The femtosecond laser-mediated procedure used OCT-guided imaging to make 12 radial laser cuts, set into the mid-periphery of the lens nucleus, that created compression joints to restore some degree of accommodation. The width of the compression joints and the diameter of the clear central optical zone varied according to the treatment arm. The total treatment zone diameter was 8.0 mm and restricted by a safety zone of 700 μm to the lens epithelium. **B**–**D** Laser lesion “scars” were noticeable in the postoperative period but started fading by postoperative month 1. OCT, optical coherence tomography

The phase 2a trial aimed to enhance near vision in patients with clear, presbyopic lenses. It involved bilateral OCT-guided femtosecond laser treatment of the lens nucleus with varying laser parameters and treatment durations. The trial evaluated improvements in AA, near visual acuity, lens optical properties, and potential complications. Although the trial did not meet its target for AA improvement, no cases of lens opacification were observed within the 12-month post-surgery period. The laser lesions gradually faded, leaving behind fine scars. Patients initially reported photopic blur and night vision issues, which diminished as the lesions faded. Notably, there were no signs of inflammation or severe complications, and visual acuity remained stable. Fig. 1

**Fig. 2:**
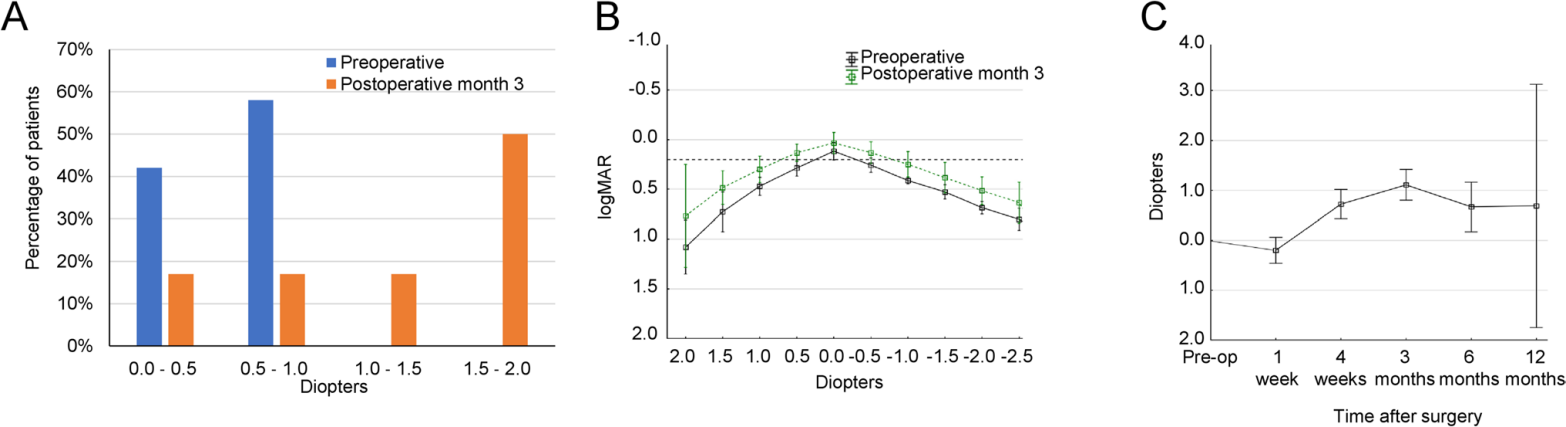
Refractive results in patients with preoperative AA of < 1.0 D following femtosecond laser treatment. **A** At postoperative month 3, the gain in AA ranged from 0.0 to 2.0 D (*n* = 12). **B** Pre- and post-operative defocus curves at month 3 (mean ± 95% confidence interval; *n* = 12). The horizontal dotted line shows the increase in preoperative to postoperative AA at logMAR 0.2. **C** Post-operative subjective refraction in patients with baseline 0.0 D refraction (mean ± 95% confidence interval). AA, accommodative amplitude

By the third month following the surgery, 40% of eyes experienced increased AA, especially those with a preoperative AA of less than 1.0 D. The defocus curve showed slight improvements at all distances. However, an unintended biomechanical effect was discovered—an average hyperopic shift of 1.0 D up to 12 months post-surgery in patients with a preoperative baseline of 0.0 D. This shift was attributed to axial lens expansion and peripheral lens thickening. Fig. 2

The findings suggest that femtosecond laser treatment holds promise in increasing AA without inducing cataracts for up to a year. However, it is important to note that a hyperopic shift may occur in some patients. Further refinement of cutting patterns and ongoing clinical research are necessary to optimize this non-invasive treatment for presbyopia.
